# Effects of the Invasive Common Myna (*Acridotheres tristis*) on Nest Site Competition and Predation in Native Birds: A Before-After-Control-Impact Study

**DOI:** 10.3390/biology14070828

**Published:** 2025-07-08

**Authors:** Iris Fortoune Engel, Ido Izhaki, Motti Charter

**Affiliations:** 1 Department of Evolutionary and Environmental Biology, Faculty of Natural Sciences, University of Haifa, Mount Carmel, Haifa 3498838, Israelizhaki@research.haifa.ac.il (I.I.); 2 Shamir Research Institute, University of Haifa, Katzrin 1290000, Israel; 3 School of Environmental Sciences, Faculty of Social Sciences, University of Haifa, Mount Carmel, Haifa 3498838, Israel

**Keywords:** invasive species, Common Myna (*Acridotheres tristis*), nesting competition, nest predation, Before-After-Control-Impact (BACI) study, nest box management

## Abstract

Despite concerns about invasive Common Mynas (*Acridotheres tristis*), few studies have experimentally demonstrated their impact on native birds. Using a Before-After-Control-Impact (BACI) design with 142 nest boxes, we assessed how Myna presence affected House Sparrows (*Passer domesticus*) and Great Tits (*Parus major*) before (2009–2010) and after (2020–2021) their arrival in 2015. Half the boxes had large entrances accessible to all three species; half had small entrances accessible only to Great Tits. In a 2022 field experiment, we manipulated nest box entrances and used cameras to study Myna predation and nest usurpation. Following the establishment of Myna, House Sparrow breeding declined by 68.1%. Great Tits increased use of small entrance boxes by 59.9% and decreased use of large entrance boxes by 45.4%. Although overall Great Tit breeding increased, fledging success declined in large entrance boxes, but not small entrance ones. In the 2022 experiment, 46% (*n* = 26) of Great Tit nests were predated; Common Mynas were responsible for 50% of these cases and subsequently bred in the usurped nests. This study experimentally demonstrates that House Sparrow breeding numbers decrease and that Great Tits change their nesting behavior after the appearance of the Common Myna. Additionally, this research found that Common Mynas impact native species by predating and usurping nests. Installing small-entrance boxes can help manage Great Tit populations. Further research should explore nest box designs for House Sparrows and their impacts on other cavity-nesting and non-cavity-nesting bird species.

## Simple Summary

The Common Myna is an invasive bird species that has rapidly spread to new areas, raising concerns about its impact on native birds. We studied how the arrival of Common Mynas in Israel affected two local bird species—the House Sparrow and the Great Tit—by observing their use of special bird nest boxes both before and after the arrival of Mynas. Some nest boxes had large entrances that all species could use, while others had small entrances that excluded the larger Common Myna. After the Common Mynas arrived, the number of House Sparrows nesting dropped by over two-thirds. Great Tits began to use the small entrance boxes more often, likely to avoid competition or harm, while their use of large boxes decreased. Although more Great Tits nested overall, fewer nestlings survived in the large boxes once Common Mynas appeared. In a separate experiment, we found that Common Mynas were responsible for half of the predations, destroying the Great Tit nests, and taking over the boxes for their own breeding. This study provides strong evidence that Common Mynas can negatively affect native birds through nest predation and usurpation, based on observed behavioral interactions and breeding outcomes, and should be carefully managed. Installing specially designed nest boxes with small entrances can help protect specific native bird species. Our findings can guide conservation efforts and nest box design to support the local bird population

## 1. Introduction

There is a strong correlation between invasive species and changes in the population dynamics of native species [1]. As invasive bird species expand into new environments, they are believed to displace or diminish native species populations by transferring diseases and parasites, hybridization, brood parasitism, predation, and competition for resources [2,3,4]. Competition for resources and predation are two main ways invasive species harm native species [5]. Native species vulnerable to predation or competition from other native species are therefore suspected to be at significant risk from invasive species [1].

Invasive species often have a negative impact on common resident species by replacing native dominant species more frequently than rare species [6]. While the needs of rare species are the focus of conservation schemes, a decline in the numbers of common species is extremely important due to their key roles in ecological functions and benefits [6,7]. For example, small-bodied native bird species are more at risk of competition for limited resources and predation by larger invasive species, as a result, smaller common birds decline faster than larger ones [7,8]. Across multiple regions, including Australia, New Zealand, and the Seychelles, the presence of Common Mynas (*Acridotheres tristis*) has been associated with declines in native bird populations. At the same time, their removal has led to measurable population recoveries [9,10,11]. Over 15 years in Israel, the invasive Common Myna population has increased by 843% [6]. During the same period, native species decline as seen with the House Sparrow (*Passer domesticus*, 28%), White-spectacled Bulbul (*Pycnonotus xanthopygos*, 44%), Graceful Prinia (*Prinia gracilis*, 35%), Palestine Sunbird (*Cinnyris osea*, 57%), Syrian Woodpecker (*Dendrocopos syriacus*, 51%), Common Blackbird (*Turdus merula*, 39%), and Eurasian Hoopoe (*Upupa epops*, 38%) [6]. Because most previous studies base their findings on correlation, there is a need for further empirical research to understand the specific mechanisms driving the decline of native species, including nest site competition and nest predation.

In areas where nest cavities are limited [12,13], invasive species may affect the breeding of native secondary cavity-nesters. Evidence is growing that as invasive bird populations increase, the populations of native cavity breeders decline. For instance, 5 out of 27 (19%) native cavity-nesting bird species in North America significantly declined after the invasive European Starling (*Sturnus vulgaris*) appeared [14]. However, empirical evidence is hard to obtain due to slow changes in native species abundance [15] and the lack of long-term pre-invasion data for reliable comparisons [16].

There are two main types of interspecific nest site competition. The first type is exploitation competition, which occurs when species interact indirectly through limited resources that become unavailable to others once occupied. The second type is interference competition, which involves direct, often destructive, and aggressive interactions between competing species, such as nest usurpation. Dominant competitor species can influence numbers and distribution by exploiting finite resources. In extreme cases, subordinate species may be absent from areas where the dominant species occupy all suitable nest sites. In moderate cases, the number of subordinate species may decline, while that of dominant species rise [12,16,17].

The larger the invasive species, the more detrimental the competition from exploitation is to the native species. Invasive species often monopolize cavity resources, as seen with Ring-necked Parakeets (*Psittacula krameri*) and Common Mynas occupying the majority of available nest sites in European and Middle Eastern urban areas [18,19]. Interference competition is likely common among cavity-nesting birds. However, this is often overlooked because documenting such interactions at the population level happens very quickly and is difficult to record, leading to a lack of experimental studies in both native species [20,21] and invasive species [20]. Aggressive interspecific interactions at nest cavities can occur before and during the breeding season, with successful pairs guaranteeing breeding opportunities. In many cases, dominant species can usurp active nest sites with egg/nestlings, causing complete breeding failure for the displaced species, similar to predation. Documenting nest usurpations is challenging because they can occur rapidly, but the consequences can have a significant impact on occupancy and breeding success at the population level [21,22,23].

Successful bird reproduction may be greatly impacted by larger bird species stealing nests. Nest usurpation and competition for cavities have been widely documented among both native and invasive species, often resulting in reduced breeding success or nest failure for subordinate species [24,25,26]. According to an Israeli experimental study native House Sparrows usurped 77.8% of Great Tit nest sites which lowers their breeding success in a manner akin to predation [27]. To prevent total nest failure, the smaller and inferior nest site competitor species may develop behavioral strategies (i.e., different breeding times or nest entrance preferences) to avoid competition for nest sites or predation [27,28,29,30,31,32].

Interactions between native and invasive cavity breeding bird species are influenced by breeding timing, nesting preferences, and cavity modification skills [19,29,33,34]. Invasive species may usurp the nests of native species with similar phenology and cavity preferences [35]. For example, in Spain, Ring-ringed Parakeets usurp cavities and sometimes kill threatened Greater noctule bats (*Nyctalus lasiopterus*) [36,37]. In North America, invasive species usurped a third of the native cavity breeders nest cavities [38]. In Ohio, invasive Starlings frequently compete with native Red-bellied Woodpeckers (*Melanerpes carolinus*), taking over 39% of their cavities [39]. In Australia, Common Mynas outcompete native parrots over nesting cavities [21]. Common Mynas are highly aggressive and frequently win interactions with native cavity conflicts, even against larger species [40].

Predation can significantly affect bird communities by reducing reproductive success [41,42]. In Brazil, 23.3% of Campo Miner (*Geositta poeciloptera*) nest failures were from predation [25]. Predation is often undocumented, as most predation occurs rapidly at all hours and is not visible, especially in cavity-breeding birds [3]. Trail cameras with triggers often miss fast-moving animals compared to those that use continuous recording video. In Spain, cavity-nesting birds reoccupied safer cavity sites with reduced predation risk than risky territories [43]. In Arizona, cavity-nesting birds gathered in shared trees as their breeding success increased, even when predation risk was higher [44]. Cavity entrance size determines which species can breed and which predators can enter [45,46].

Invasive predators have facilitated species extinction and decline [47]. Invasive species pose a serious threat to mainland and native endangered species, particularly on islands, through predation [5]. For instance, a large proportion of Black Robin (*Petroica traversi*) nests in New Zealand were predated by invasive European Starlings [48]. Anecdotal evidence suggests that Common Myna preys on eggs and reptiles in seabird colonies [49,50,51], the prevalence of predation in cavity-breeding species remains unclear, and the effect of invasive species on native birds is probably underreported [8].

This study has two parts. The first part aims to quantify the impact of the invasive Common Myna on the breeding of two native secondary cavity-nesting species, Great Tits and House Sparrows, using a Before-After Control Impact (hereafter BACI) both before (2009–2010) and after (2020–2021) the Common Myna’s first appearance in 2015. We used two different sizes of nest box entrances: one that only Great Tits can enter and another that both larger species (House Sparrows and Common Myna) can also access to determine whether the breeding of native cavity-nesting species changed after the appearance of the Common Myna. The study hypothesizes that after the appearance of Common Myna, the native species will decrease their breeding attempts, resulting in reduced breeding success due to exploitation and interference with nest site competition. We also predict behavioral changes by Great Tits that will prefer nest sites that exclude Common Mynas. Additionally, we predict that the breeding numbers of House Sparrows will decrease due to direct competition with Common Mynas and the lack of available nest sites that Common Mynas cannot enter.

The second part was an experimental study that manipulated nest box entrance sizes and used continuous recording video to determine whether Common Mynas predate and usurp (through interference competition) Great Tit nests. We hypothesize that Common Mynas will have high rates of predation and usurpation. Furthermore, this study provides important recommendations for mitigating the effects of Myna invasion on native birds.

## 2. Materials and Methods

### 2.1. Study Site

The study site is located in the village Moshav Ram On (30 km south of Nazareth) in the south-east part of the Jezreel Valley, Israel (32°31′55″ N, 35°15′25″ E), 80–90 m above sea level, with a semi-arid climate. The 1.7 km^2^ study site hosted 984 residents in 236 houses (1–2 floors) on a 500 m^2^ plot, including a public garden [52]. The village is surrounded by agriculture, mainly fields of sweet corn, alfalfa, oats, wheat, grape vines, almond plantations, and olive groves.

### 2.2. Studied Species

This study examines the breeding of three Passerine cavity breeders: two native species (House Sparrow and Great Tit) and one invasive species (Common Myna). All three are secondary cavity breeders found near human habitats [53]. The Great Tit, is the smallest species of the study at 15.5 g and a length of 14 cm, is a very common resident in the Mediterranean region of Israel. Great Tits inhabit primarily areas with trees and bushes, its diet combines insects, spiders, fruits, and seeds. Great Tits lay clutches of 5–7 eggs in either natural or artificial cavities during January to June [54].

The House Sparrow is native species smaller than the Common Myna but larger than the Great Tit, weighing 24 g and measuring 17 cm in length. In Israel, house sparrows are common inhabitants found near human settlements. They eat insects, fruits, plants, cereal seeds, and leftover food from people. From March to August, house sparrows nest exclusively in cavities in Israel, laying clutches of three to six eggs and producing two to four clutches annually in nest boxes, natural tree holes, and other cavities [55].

Originating in South Asia and the Indian subcontinent, the Common Myna was brought to Israel in the late 1990s after escaping or being released from a Tel Aviv zoo [56]. Currently one of the most prevalent birds in Israel, Common Mynas are also becoming more widespread in the Middle East [6,57]. The Common Myna is considerably larger (120 g and 25 cm) than the native species examined and was first observed in this study area in 2015. To estimate the abundance of Common Mynas during the breeding season, we conducted 20 focal point surveys across the study site from 7 March to 26 July 2021. Each survey recorded the number of Mynas observed, with an average of 32 individuals per survey (range: 15–59). While these counts offer an indicator of local abundance, we did not gather detailed density data, such as breeding pair or territory mapping.

The nests of the three secondary cavity breeders are distinguishable by their shapes and materials. Great Tits build well-organized nests using weeds, moss, and soft linings made from animal fur and hair. House Sparrows create nests with dried grass, weeds, and feathers in a disorganized manner. Common Mynas accumulate broad leaves, feathers, plastic bags, other human waste, and occasionally snake skin.

### 2.3. Before-After-Control-Impact (BACI)

**Figure 1:**
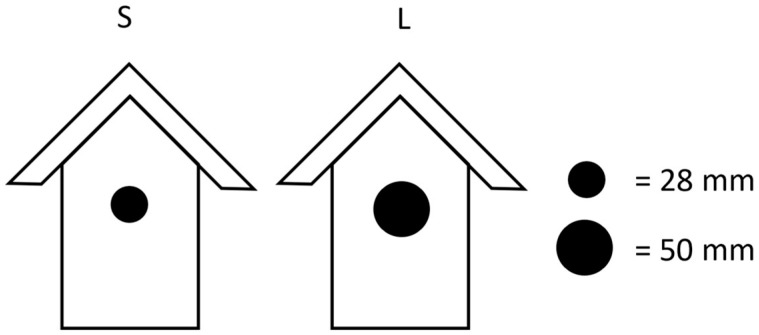
Two types of nest box entrances with different sizes entrances were used for experimental manipulation: Small entrance (2009 = 61 nest boxes, 2010 = 69, 2020 = 68, 2021 = 62) nest boxes allowed access only for the smaller Great Tits, whereas the large entrance (2009 = 60 nest boxes, 2010 = 73, 2020 = 64, 2021 = 75) allowed access for Great Tits and also the larger House Sparrows and Common Mynas.

This study used a BACI design to assess the impact of Common Myna presence on native breeders. We compared the occupation and breeding success of Great Tits and House Sparrows in nest boxes during 2009–2010, before the Common Myna’s arrival [28], to 2020–2021, after the Common Myna’s appearance. The nest boxes were made from untreated plywood (15 cm × 15 cm × 24 cm) and were placed 40–50 m apart on large trees at 1.5–2 m height. From 2010 to 2019, many of the nest boxes were destroyed, and new boxes were added in the same locations a year before the study (2019). Nest boxes with two different entrance sizes (small entrance = 28 mm and large entrance = 50 mm) were used. Great Tits could enter both sizes, whereas House Sparrows and Common Mynas could only enter the larger 50 mm entrances (Figure 1). 121 boxes were installed in 2009 (61 small and 60 large), 142 in 2010 (69 small and 73 large), 132 in 2020 (68 small and 64 large), and 137 in 2021 (62 small and 75 large). The variation in the number of nest boxes between years was primarily due to the destruction of boxes. During the earlier study [28], we used two entrance sizes to examine competition between Great Tits and House Sparrows. Fortunately, we utilized a 50 mm entrance size, which also allows the Common Myna to enter, now that they have appeared in the same study since 2015, giving us the opportunity to conduct a BACI study. All nest boxes had an internal entrance size of 50 mm. However, the external entrance holes could be adjusted to either 50 mm or 28 mm using a small metal restrictor plate screwed into the front. Consequently, there was no difference in the internal height of the entrance holes from the bottom of the nest, allowing nestlings to reach the entrance holes of both types similarly. To prevent pseudoreplication, entrance sizes were switched between small and large nest boxes between the first and second breeding seasons in both periods (Figure 1).

Nest boxes were monitored twice weekly from January to August over all four breeding seasons. We recorded all breeding attempts, and nests were identified by species (Great Tits, House Sparrows, or Common Myna). Breeding data were collected as per [28]: nest built, clutch size (number of eggs), brood size (number of nestlings at hatching), and number of fledglings. Clutch sizes were sometimes unobservable due to the presence of incubating females (we did not flush them to count). Nests were not removed during the breeding season but were cleaned between seasons. We documented the percentage of nest boxes that Great Tits built nest in, the percentage of nest boxes that great tits laid clutches, and the percentage of pair that succeeded in fledging at least one nestling. The breeding parameters of the Great Tit were compared, both before and after the appearance of the Common Myna at the study site, between nest boxes with small and large entrances. The latter are more exposed to potential threats, such as predation and nest site competitors.

There were no specific changes in weather and the habitat before (2009–2010) and after (2020–2021) the appearance of the Common Myna. Specifically, using a generalized linear mixed model with year as a random variable, no significant differences were found in rainfall (F_1,30_ = 0.30, *p* = 0.586), number of rain days (F_1,30_ = 1.819, *p* = 0.187), maximum daily temperature (F_1,30_ = 0.022, *p* = 0.883), and minimum daily temperature (F_1,30_ = 0.32, *p* = 0.859) between the periods before and after the appearance of the Common Myna (Israel Meteorological Center, 2023). The number of residents in the village remained relatively stable, and no major construction occurred at the study site between 2009 and 2021.

### 2.4. Do Common Myna Predate and Usurp Great Tit Nests? Field Experiment

**Figure 2:**
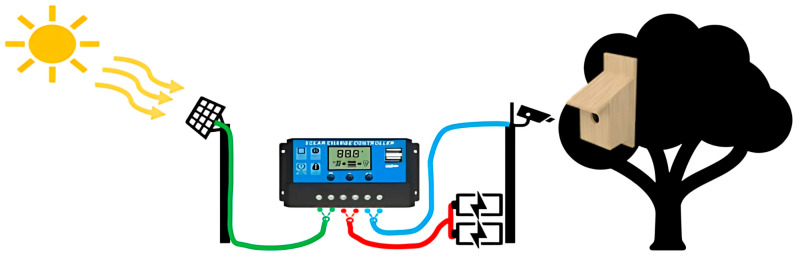
The surveillance solar camera system consists of two 12V22AH/20HR batteries housed in a plastic box with a stand, a 1.5 m metal pole, a 2 m wooden pole, a solar charge controller (rated voltage: 12 V/24 V), a 30 W solar panel (35 × 65 cm), an EXIR MINI BULLET surveillance camera (Model: DS-2CD2021G1 4 mm B, Hikvision), a 128 GB Sandisk Ultra memory card, and a flexible multicore cable (3182Y, H05VV-F, unscreened, 2 core, 1.5 mm^2^).

**Figure 3:**
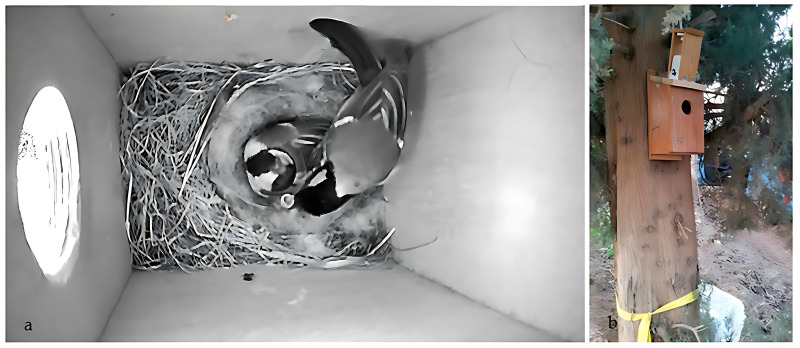
Surveillance solar continuous video recording system for filming Great Tits nesting. An example of what you see inside (**a**) and how the camera fits onto the top nest box (**b**).

During the 2022 breeding season, a field experiment was conducted involving all nest boxes (*n* = 142), initially with small entrances (only Great Tits could breed) at the start of the season. Once Great Tit females had completed egg-laying and had incubated the eggs for 5–7 days, the entrances of half of the active nest boxes were enlarged by removing a metal plate, thereby converting them to large-entrance nest boxes. At that time, surveillance solar cameras (EXIR MINI BULLET, Hikvision) (Figure 2 and Figure 3) were added and placed on the box lid to film the inside. We installed cameras and enlarged the entrances 5–7 days into incubation to minimize the risk of nest abandonment, which is highest during the early egg-laying phase. Had we enlarged the entrances at the time of the first egg, the likelihood of abandonment would have been significantly higher. Indeed, if it had been possible to enlarge the entrances immediately after the first egg was laid, we might have observed even more cases of predation. Of the 52 nests filmed, there was no significant difference in clutch size (Mann-Whitney Z = −0.115, *p* = 0.91) or laying date (Mann-Whitney Z = −0.075, *p* = 0.94) between nests with small (*n* = 26) and large (*n* = 26) entrance nest boxes. Surveillance solar cameras were placed inside all 26 small and 26 large entrance nest boxes to document interactions (predation, usurpation, etc.) with other species. Since it doesn’t rain much in Israel, we had no issue using solar panels with batteries even during overcast weather. The camera continuously recorded the inside of the box during the day and night, using 1280 × 720 pixel footage, which was sufficient for species identification and observing behavior. Configuration was done using a laptop, SADP software version 3.0.2.4, and network cable. Data was stored on 128 GB Sandisk Ultra memory cards and was changed every 3–7 days. We filmed until nesting was over by fledging or death. The primary goal was to determine whether the nest was predated or usurped, and if so, which species did it, in order to establish whether they predated all or only part of the nest (egg/nestlings). Nest interference was defined as a pair that usurped the nest, and predation was defined as when another species predated eggs or nestlings.

### 2.5. Statistical Analysis

We used Generalized Linear Mixed Models (GLMMs) with a binomial error distribution and a logit link function to analyze nest box occupancy, clutch laying, and breeding success of House Sparrows and Great Tits in relation to nest box entrance size and the presence of Common Mynas. All GLMMs included nest box identity as a random effect to account for repeated measures and spatial non-independence. Separate GLMMs were constructed for each of the following response variables: (1) nest box occupancy (presence/absence of a breeding attempt), (2) clutch laying (presence/absence of egg-laying), and (3) breeding success (successful fledging of at least one nestling). Fixed effects included time period (before vs. after Common Myna establishment), nest box entrance size (small vs. large), and their interaction, where relevant. When applicable, analyses were also performed separately for each entrance size category to assess within-category temporal changes. For all GLMM analyses, we reported odds ratios (OR) and 95% confidence intervals (CI) by exponentiating the model coefficients to aid interpretation of effect sizes. Model diagnostics were performed, including checks for overdispersion and visual inspection of residuals to assess model fit. These steps revealed no significant violations of model assumptions.

For the field manipulation nest entrance size experiment, we used a two-way 2 × 2 Chi-square (χ^2^) to determine whether the number of pairs that succeeded in fledging at least one young varied. All analyses (GLMMs and χ^2^) used SPSS version 27 (IBM Corporation, Armonk, NY, USA).

## 3. Results

### 3.1. Myna Impact on House Sparrow (BACI)

**Figure 4:**
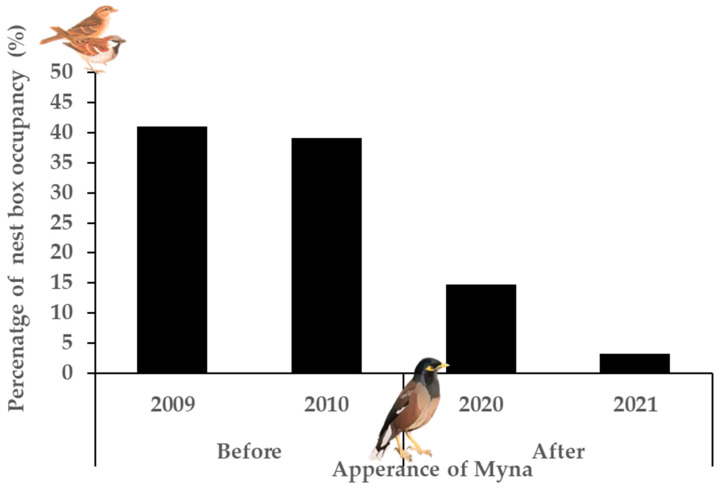
Nest box occupancy by House Sparrows in large entrance before (2009–2010) and after (2020–2021) the appearance of Common Myna.

House Sparrows occupied significant less large entrance nest boxes after the appearance of Common Myna (F_1,258_ = 13.33, *p* < 0.001; OR = 0.253, 95% CI [0.12, 0.53]) (Figure 4). There was a 68.1% decrease in the number of nest boxes occupied by House Sparrows before (n_2009_ = 25 pair, n_2010_ = 27) and after Common Myna (n_2020_ =10, n_2021_ = 2) appeared. After the appearance of the Myna, during the 2020–2021 breeding seasons, none of the House Sparrows successfully fledged young, and there were significantly fewer attempts to breed in the second year (2021) (χ^2^ = 5.35, df = 1, *p* = 0.02). This is most likely because the House Sparrows that attempted to breed did so in nest boxes for the first time since the new nest boxes were added in 2019, following the destruction of the previous nest boxes from 2010 to 2019.

### 3.2. Myna Impact on Great Tits (BACI)

**Figure 5:**
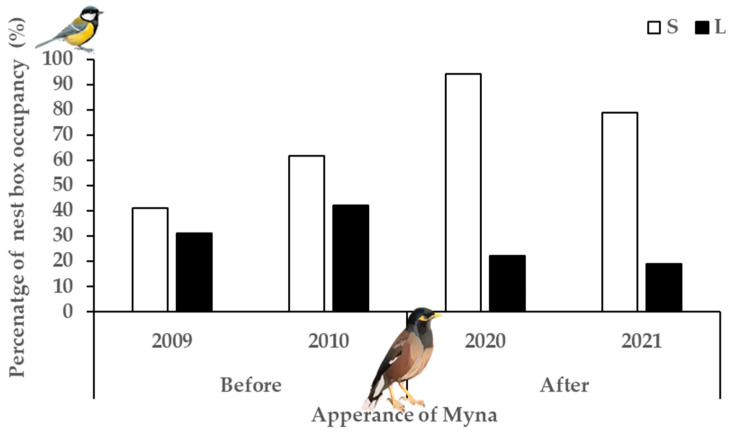
Percentage of nest boxes that Great Tit built nests in small (white) and large (black) entrance nest boxes before (2009–2010) and after (2020–2021) the appearance of Common Myna.

We compared the number of Great Tit nests built both before and after Common Myna appeared and in the two different nest box entrance sizes (small vs. large) (F_2,529_ = 42.56, *p* < 0.05). More Great Tits nests were built after the establishment of Common Myna (F_1,529_ = 5.10, *p* < 0.05; OR = 1.540, 95% CI [1.06, 2.24]) (Figure 5). Furthermore, Great Tit pairs built more nests in small entrance nest boxes than in large entrance nest boxes (F_1,529_ = 82.68, *p* < 0.001; OR = 0.175, 95% CI [0.12, 0.26]). Using only data from large entrance nest boxes, great tits occupied significantly lower percentage (45.4% less) of large entrance nest boxes after the arrival of Common Myna (F_1,258_ = 8.51, *p* < 0.01; OR = 0.43, 95% CI [0.24, 0.76]). In comparison, in a similar GLMM analysis using only data from the small entrance nest boxes, we found that Great Tit occupied more small entrance nest boxes (59.9% increase) after the appearance of Common Myna (F_1,270_ = 32.94, *p* < 0.001; OR = 5.51, 95% CI [3.07, 9.90]) (Figure 5).

**Figure 6:**
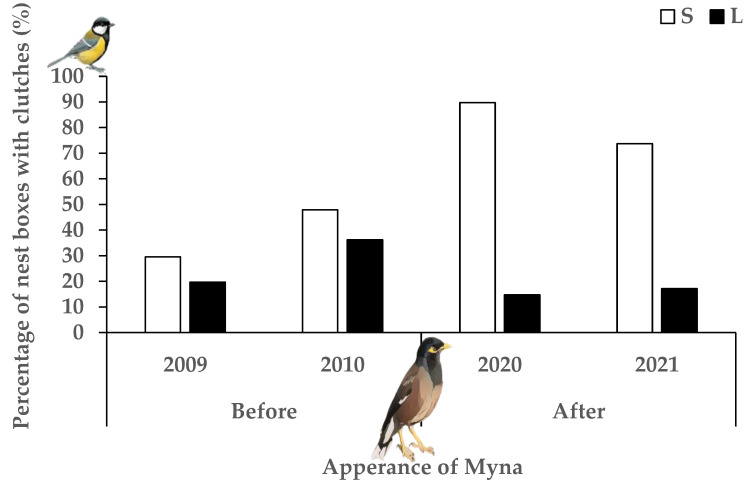
Percentage of nest boxes that Great Tit laid clutches in small (white) and large (black) entrance nest boxes before (2009–2010) and after (2020–2021) Common Myna establishment.

We compared the percentage of nest boxes that Great Tit laid clutches during the two periods (before and after Common Myna presence) and in both of the nest box entrance sizes (small versus large) (F_2,529_= 42.37, *p* < 0.001), with nest box as a random variable. There was significant increase in the percentage nest boxes that Great Tits laid eggs after the appearance of Common Myna (F_1,529_ = 12.64, *p* < 0.001; OR = 2.01, 95% CI [1.37, 2.95]) (Figure 6) and there were more Great Tits pairs that laid eggs in small entrance nest boxes compared to large entrance nest boxes (F_1,529_ = 78.57, *p* < 0.001; OR = 0.17, 95% CI [0.12, 0.25]). Using only data from the large entrance nest boxes, we found that great tits laid significantly less (48.1% less) clutches after the appearance of Common Myna, (F_1,258_ = 7.03, *p* < 0.01; OR = 0.43, 95% CI [0.23, 0.81]) Conversely, in a similar GLMM analysis using data only from small entrance nest boxes, we found that great tit laid in a higher percentage of small entrance nest boxes (96.8% more) after the appearance of Common Myna (F_1,270_ = 43.51, *p* < 0.001; OR = 6.27, 95% CI [3.62, 10.83]) (Figure 6).

**Figure 7:**
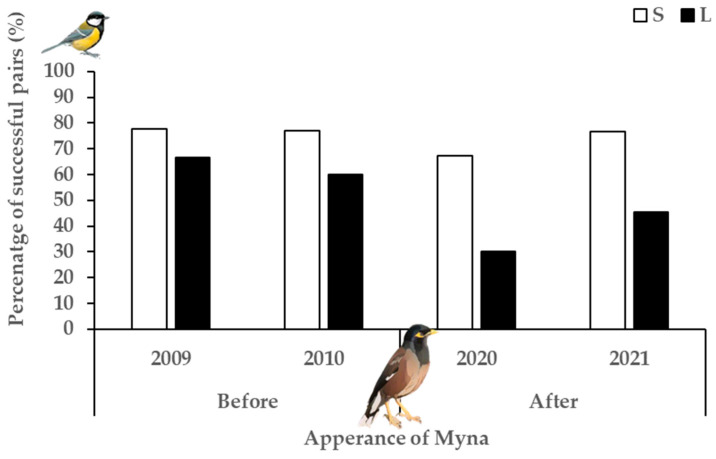
Percentage of Great Tit’s successful breeding attempts (*n* = 227 breeding attempts) fledged at least one nestling) in small (white) and large (black) entrance nest boxes before (2009–2010) and after (2020–2021) the appearance of Common Myna.

We compared the percentage of active (laid at least one egg) Great Tit pairs that succeeded in fledging at least one nestling during before and after Common Myna’s appearance and in the two nest box entrance sizes (small versus large) (F_2,218_ = 5.67, *p* = 0.004). Our results indicate that the Great Tit succeeded to fledge in more small than large entrance nest boxes (F_1,218_ = 10.78, *p* < 0.001; OR = 0.32, 95% CI [0.16, 0.63]) and the percentage of Great Tits that fledged young decreased after the appearance of Common Myna (F_1,218_ = 3.80, *p* = 0.05; OR = 0.53, 95% CI [0.27, 1.01]) (Figure 7). Using data only of large entrance nest boxes, with nest box as a random variable, we found a lower percentage of great tits succeeded to fledge at least one young (36.4% decrease) after to the appearance of Common Myna (F_1,54_ = 4.47, *p* < 0.05; OR = 0.28, 95% CI [0.09, 0.94]). Conversely, in a similar GLMM analysis considering only at small entrance nest boxes, great tit succeeded to fledged nestlings similarly between the two periods (F_1,163_ = 0.76, *p* = 0.38; OR = 0.70, 95% CI [0.32, 1.56]) (Figure 7).

### 3.3. Do Common Myna Predate and Usurp Great Tit Nests?

During the 2022 field experiment in which we added cameras inside the nest boxes, 21 pairs of Great Tits successfully fledged young from the small entrance nest boxes, while five pairs did not. In contrast, 12 pairs succeeded in the large entrance nest boxes during the same year, while 14 pairs failed. A significantly higher number of Great Tit pairs failed to fledge at least one young in the large (53.8%) entrance nest boxes compared to the small (19.2%) entrance nest boxes (χ^2^ = 6.72, df = 1, *p* = 0.01).

**Figure 8:**
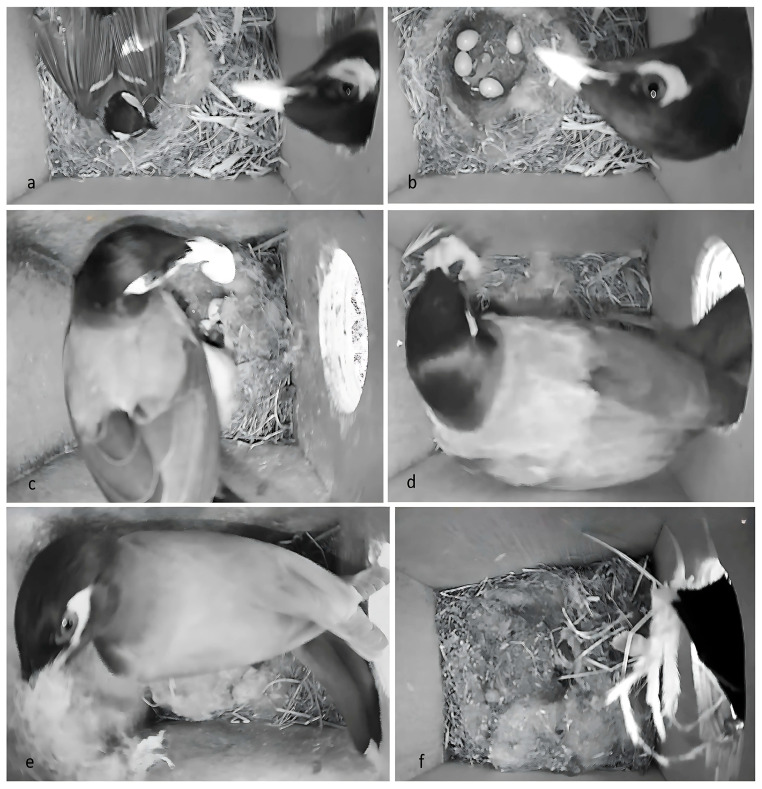
(**a**) A Common Myna invades a nest box, disturbing an incubating Great Tit female. (**b**) Following the forced departure of the Great Tit female, abandoning her eggs and a newly hatched nestling, the Common Myna enters the nest box and preys on the Great Tit’s (**c**) eggs and (**d**) nestling. (**e**) The Common Myna removes the Great Tit’s nesting materials from the nest box after predation on the eggs. (**f**) The Common Myna adds its own nesting materials over the Great Tit’s active nest, while a nestling remains present.

In the large entrance nest boxes, 14 nests failed, with predation accounting for 12 of these failures (46.2% of the Great Tit nests). In contrast, five nests in the small entrance nest boxes, the adults abandoned their clutch for unknown reasons. Among the 12 predated nests, 50% (*n* = 6) were predated by Common Mynas, 40% (*n* = 5, Figure 8) by Syrian Woodpeckers (*Dendrocopos syriacus*), and 10% (*n* = 1) by Eurasian Jays (*Garrulus glandarius*).

Common Mynas usurped nests while Great Tit females were inside the nest box, causing them to abandon their nests in five instances, and afterward preying on the eggs (*n* = 4) and nestlings (*n* = 1) (Figure 8). In another case, Great Tit parents were forced to abandon their nest due to a Common Myna that started bringing nesting material inside a Great Tit nest with nestlings, causing the pair to abandon the nest and the nestlings to die of starvation. In all six instances where Common Mynas usurped the nest boxes, they built their nests on top of the Great Tit nests within two weeks.

Overall, 50 Great Tit eggs and 10 nestlings were predated. Of those, Common Mynas predated 27 eggs and 1 nestling, woodpeckers 23 eggs and 3 nestlings, and Jays predated 6 nestlings. Common Mynas predated all eggs and nestlings in the nest boxes, either during a single event (*n* = 2) or over two sequential events (*n* = 3). In contrast, woodpeckers often left some eggs and nestlings in the nest during a single predation event, killed the incubating female Great Tit (*n* = 2) and did not use the nest boxes for breeding.

## 4. Discussion

### 4.1. Impact on House Sparrows (BACI)

The BACI results effectively quantify the negative impacts of the invasive Common Myna on native breeders by focusing on their interaction with the limited resources of nesting cavities. The results reveal that the Common Myna affects the two studied native species differently. During the study period, the House Sparrow population was significantly affected, with breeding attempts dropping from 40.0% (2009–2010) to 12.8% (2020–2021) five years after the arrival of the Common Myna. Furthermore, during 2020–2021, the number of breeding attempts by House Sparrows decreased from 10 (14.7% of nest boxes) to just 2 (3.1% of the nest boxes) breeding attempts between the two years, and none of the 12 breeding attempts successfully raised young. Not only were there fewer pairs after the Common Myna appeared, but there was also a decrease in the number of breeding attempts between the two breeding seasons following the reestablishment of large entrance nesting boxes in 2019. This was most likely due to the pairs failing to fledge young because of the Common Myna. Typically, birds do not nest in the same site after failing to breed the previous year [12,58,59]. A study built on a survey of observations in Israel [6] found that 15 years after Common Myna appeared, House Sparrow numbers decreased by 28%, consistent with the decreased breeding numbers in this study.

Unlike Great Tits, House Sparrows lacked nest boxes that were protected from Common Mynas. While the BACI results show that nest-site competition plays a central role in the decline of House Sparrow breeding numbers after the arrival of Common Myna, this study did not have a nest box design that House Sparrows could enter but would exclude Common Mynas. Unfortunately, we did not have a specially designed nest box for House Sparrows because the invasion of Common Mynas was not anticipated during the initial setup in 2009–2010, when competition for nest sites between House Sparrows (which are larger than Great Tits) and Great Tits was studied. Direct competition is likely between House Sparrows and Common Myna since both species are secondary cavity nesters that breed in similar nest sites. However, since we did not have a nest box that allowed sparrows but blocked mynas, it is impossible to isolate the specific contribution of nest-site limitation in the House Sparrows’ decline from other potential competitive interactions, such as interference or exploitative competition for food resources. This may be relevant as House Sparrows and Common Mynas primarily forage on the ground. Native and invasive species have been found to compete not only for nest sites [60] but also at feeding sites, as found by invasive ring-necked parakeets outcompeting native starlings [61]. Native birds have been found to reduce feeding rates and increase vigilance in response to invasive species [62]. There is a need to study whether providing protected nest sites (entrances that House Sparrows can enter but Common Mynas cannot) will help determine if the decrease in House Sparrows is due to a lack of protected nest sites and/or other factors, such as competition for food and feeding sites. The two species most likely compete for both nest sites and food through exploitation and interference competition, intensifying the negative effects and explaining the significant decline in House Sparrow numbers in Israel [6].

While the results suggest that the decline in House Sparrow breeding was linked to the appearance of the Common Mynas, other factors may also contribute to the population decrease. For example, decreases in food supply or changes in microhabitat quality could impact House Sparrow populations, either independently or together with competition caused by Myna. Both species forage on the ground and may compete for anthropogenic food sources, potentially intensifying interspecific interactions. Although no significant changes in weather or human population were detected during the study period, future studies should investigate the role of food resource competition and urban landscape changes in shaping House Sparrow dynamics [61,62].

The magnitude of the observed impacts on native species likely depends on the local density of Common Mynas. Higher densities may increase competition for nest sites and predation pressure, especially in areas with few cavity options. Although our BACI design effectively captures presence or absence effects, the absence of detailed Myna density data limits our ability to model density-dependent responses or apply the findings to other regions. Future research with spatially explicit density estimates could help identify impact thresholds and inform targeted management strategies.

### 4.2. Impact on Great Tit (BACI)

The Great Tit was found to be a subordinate competitor for nest sites against House Sparrows before the arrival of the Common Myna [27,28], and it changed its nest selection behavior after the Common Myna’s establishment. Specifically, the Great Tit increased its occupation in small entrance cavities for breeding by 59.9%, which prevents the Common Myna from entering, and reduced its use of large entrance cavities accessible to Common Mynas by 45.4%, most likely as an antipredator and anti-competitor strategy (interference competition). Likewise, Great Tit also had a 96.8% increase in clutches laid in small entrance nest boxes and a 48.1% decrease in large entrance nest boxes after Common Myna’s appearance. Furthermore, after the presence of Common Myna, Great Tits succeeded in fledging young more in the small entrance cavities (72.1%) compared to large entrance cavities (39.7%) after the Common Myna invasion. Overall, the breeding success of Great Tits decreased after the Common Myna appeared, with a significant drop in large entrance nest boxes. Before the Common Myna’s presence, Great Tits occupied both nest site sizes roughly equally in the beginning of the breeding season and later in the breeding Great Tit bred less in the large entrance nest boxes to reduce competition with the larger House Sparrows [28]. The Japanese tit (*Parus minor*) also was found to breed more frequently as an anti-predatory strategy in nest sites that prevent predators from entering, which also influences their nest defense behavior [63].

The combination of have a natural nest site competitor, House Sparrows, that can usurp Great Tit nest sites [27], with an even larger more aggressive invasive nest site competitor (Common Myna), may have forced Great Tits to avoid breeding altogether in the large entrance nest sites and to select nest sites that prevent both nest site competitors from entering. The Great Tit’s preference for nests with small entrance sizes indicates an adaptive behavioral response to increased competition and predation from the Common Myna. The increased use of small entrance nest boxes following the arrival of the Common Myna may indicate phenotypic plasticity [64], a flexible behavioral response to avoid competition and nest usurpation. Alternatively, if such shifts are heritable and last across generations, they could suggest early-stage selection favoring individuals that select safer nesting sites. Although our study design cannot differentiate between these mechanisms, the quickness of the shift emphasizes the potential for behavioral adaptation when faced with new ecological threats. Great Tits and other birds are under evolutionary pressure to maximize breeding success, which includes choosing nest locations that minimize competition and predation. Over time, invasive species, such as Common Myna and Ring-necked Parakeet, occupy most cavities, leaving few for native species [18,65]. The Great Tit’s susceptibility to the larger invasive Common Myna, much like that of House Sparrows, is responsible for the significant decline in reproductive success in large entrance cavities.

Interestingly, as an unexpected outcome given the anticipated negative effects of competition, the number of breeding pairs increased after the arrival of the Common Myna, primarily in the small entrance nest boxes. Although more pairs bred, the proportion of those that successfully fledged young decreased, primarily due to failures in large-entrance boxes. Since the number of nest sites/boxes remained similar between the periods, this shift likely indicates changes in nesting behavior rather than increased opportunity. The decline of House Sparrows and the appearance of the Common Myna, a larger nest site competitor, may have forced Great Tits to shift toward small-entrance boxes, thereby reducing both interspecific competition and the energetic costs associated with defending or occupying larger boxes. In the past, Great Tits were found to prefer breeding in nest boxes with larger entrances in the fall [28], before House Sparrows would breed. By breeding in the safer small entrance nest boxes after the arrival of the Common Myna, due to their inability to compete with the larger Myna and potentially due to the fear of predation, Great Tits may have experienced improved breeding because of the reduced energy they would have spent fighting House Sparrows, leading to more pairs succeeding and an increase in population. Unfortunately, we lack comparative data from other regions to confirm whether this trend is consistent elsewhere.

While the BACI design suggest that the Common Myna contributed to declines in House Sparrow breeding and altered Great Tit nesting behavior, we acknowledge that ecological systems are complex and can be influenced by multiple interacting factors. Even though we did not find significant changes in weather patterns (rainfall, temperature, or rain days) or habitat structure (e.g., urban development, human population size) between the pre- (2009–2010) and post-Myna (2020–2021) periods, we cannot fully exclude the influence of other co-occurring factors such as subtle shifts in microhabitat quality, predator dynamics (e.g., Syrian Woodpeckers, Eurasian Jays), or food resource competition. These may have interacted with Myna impacts to shape native bird responses and warrant further investigation.

### 4.3. Predation and Nest Usurpation (Interference Competition)

This experimental study demonstrated that Common Mynas usurp nests (through interference competition) and prey on their eggs and nestlings. In the large entrance boxes, 23.1% of the Great Tit breeding attempts failed due to predation by Common Mynas and interference competition. Overall, Common Myna were responsible for 50% of the Great Tit nests predated. In every instance, Common Mynas quickly started building nests in the nest boxes. This nest usurpation rate from this experimental study aligns with observational studies on invasive species. For example, across the United States and Canada, a study found that nearly one-third of 871 observations involved an invasive species that has taken over a native species’ nest box [38]. In another study, exploitation competition was found as 78% of nest boxes were occupied by Common Mynas, leaving limited available nest sites for Great Tits and other native species [18]. These studies demonstrate that the combination of exploration and interference competition by invasive species can alter the population dynamics of native species.

Great Tit nest predation by Common Mynas occurred in 19.2% of the sampled breeding attempts in large entrance cavities. This rate is consistent with other cavity-breeding birds, such as the Campo Miner [25]. Similarly, invasive European starlings’ nest predation of the black robin an endangered open nest breeder, was the most significant cause of nest failure (20.6%) [48]. In an experiential study, European Starling were also found to usurp Great Tit nests [20]. Like the invasive European Starling [66], the Common Myna, like both species, does not occupy limited cavities (only exploitation competition), and nest predation disturbs, predates, and usurps nests primarily during the early stages of nesting. All nest predations by Common Mynas occurred early in the breeding season, while most Great Tits were incubating eggs or young nestlings. Unlike other studies [67], no partial nest failures were observed; every predation event led to complete nest abandonment and breeding failure.

Common Mynas’ aggressive behavior includes chasing away incubating females and predating on eggs and nestlings. Other predators, such as the Syrian Woodpecker and the Eurasian Jay, contribute to nest failures, although to a lesser extent than the Common Myna. The combined pressure from native and now invasive predators decreases the ability of native cavity breeders to breed, thus stressing the complex predator-prey interactions in ecosystems affected by invasive species. In addition to direct predation, Common Myna activities, such as aggressive displacement of adults or increased noise and disturbance around nest sites, could indirectly facilitate predation by native predators like the Syrian Woodpecker and Eurasian Jay. For example, altered parental behavior (e.g., reduced nest attendance or delayed feeding visits) in response to Myna aggression might expose nests to higher predation risk. For example, the invasive predator Caspian gull (*Larus cachinnans*) caused complex direct and indirect effects on the native waterbird, resulting in changes to the spatial distribution of native nests and altering the predation rate by native predators [68]. However, it is also possible that these predators act independently. In another study, female sex-specific survival of the Seychelles warblers (*Acrocephalus sechellensis*) was found to be lower due to Common Mynas inflicting severe injuries to incubating females [69]. There are concerns that the Common Myna may prey not only on nest cavities but also on open-nesting birds.

Common Mynas compete with native cavity-nesting birds through interference competition (nest usurpation and predation) (This study), and previous studies in Israel have shown that they also engage in exploitation competition (monopolizing limited nest cavities) [18]. The BACI results show that House Sparrows experienced a sharp decline in breeding attempts and success after the arrival of Mynas, but since there were no nest boxes protected from Myna, we are unsure whether the cause was exploitation and/or interference competition for nest sites and/food. In contrast, Great Tits changed their nesting behavior to small-entrance boxes, probably to avoid direct interference from Common Myna, but pairs nesting in large-entrance nest boxes experienced lower fledging success due to interference competition. The presence of a large invasive species that both exploits limited nest sites and preys on or usurps nests may explain the decline in native bird populations [6].

### 4.4. Implications for Management and Conservation

Given the Common Myna’s preference for human-dominated habitats and its broad diet, the species significantly threatens urban diversity [10,70]. This study has shown that specialized nest boxes with entrance sizes that prevent invasive species, such as the Common Myna, from entering can be effectively used not only to increase the number of nest sites but also as a management tool to protect Great Tits. There is high importance of well-designed nest box programs for management and conservation [29,71,72,73,74]. Even though the population of common myna has increased, more great tit pairs were able to breed due to the presence of nest boxes that prevented the former from entering. In comparison, populations of House Sparrows without a specially designed nest box decreased during the same period. This study demonstrates how proper management efforts can reduce the impact of invasive activities on specific native species. These findings highlight the need for an active monitoring program of native breeding bird populations and adaptive management strategies to lessen the negative impacts of invasive species on native flora and fauna.

While adding nest boxes can be an effective tool for protecting some native cavity breeder birds, the addition of additional nesting cavities may also lead to unintended consequences if not carefully designed and monitored. For instance, nest boxes may attract predators or be monopolized by invasive species, thereby intensifying the pressures they aim to reduce. Additionally, increasing nest box availability without controlling invasive species populations could unintentionally benefit the invaders. To mitigate these risks, we recommend implementing nest box programs within an adaptive management framework. This approach involves conducting experimental trials, systematically monitoring occupancy and breeding success, and making iterative adjustments based on observed results.

Although our study highlights the significant impact of Common Mynas in a Mediterranean semi-arid village, the extent to which invasive cavity breeders affect native birds likely varies across different biomes and levels of urbanization. For example, in urban and island ecosystems, where natural cavities are often scarce, competition for nesting sites is more intense, whereas in forested or rural areas with abundant nesting resources, the impact may be less severe. [75]. Additionally, areas with multiple invasive cavity breeders might face combined pressures on native species. Cross-site studies are necessary to explore how ecological context influences the severity of invasive species effects.

Removing invasive plants, fish, or invertebrates seldom incurs opposition, but managing birds and mammals tends to be more controversial [76,77]. There are currently no Common Myna removal programs in Israel, which have been found to decrease predation and increase breeding success [9,78,79,80,81]. One of the reasons there are no removal projects is that people in Israel are against all forms of culling. Most people in the public lack knowledge of the effects of invasive species due to a lack of scientific studies and video documentation of predation/ nest usurpation available to the public [82]. A study in the United States and Canada found that people who observed an invasive species taking over a native species positively related to the environmental management actions they took. [38]. Witnessing predation can trigger disgust and anger [83]. Using content (video/ pictures) of Common Myna predating native wildlife can raise public awareness and encourage management activities [38]. Furthermore, many trapped invasive animals are housed in expensive long-term facilities, diverting funds from more effective conservation efforts such as preventing collisions with glass or power lines. However, these practical measures receive less media coverage than stories of “rescued” animals. Enhancing public education and reducing excessive anthropomorphism are essential.

## 5. Conclusions

Our findings demonstrate the vulnerability of secondary cavity-nesting birds to competition and predation by invasive species and that Common Myna is a clear driver of native species decline rather than a passive passenger of environmental change [84]. This study provides strong experimental evidence of the impact of invasive Common Myna on native cavity nesters through nest usurpation and predation, and suggestive evidence of broader population-level effects based on long-term breeding trends. The presence of Common Mynas led to a sharp decline in House Sparrow numbers, forced Great Tits to alter their nest site selection to avoid competition and predation, and also provides evidence that Mynas act as drivers of native species through direct nest usurpation and predation. These results show the global risk invasive secondary cavity nesters pose to native birds in locations where nesting sites are limited.

Management strategies are necessary to reduce the negative impacts of invasive species. Using nest boxes with small entrances that exclude Common Mynas was associated with increased fledging success in Great Tits, suggesting they may help mitigate some negative impacts of Myna presence. Additional measures are necessary to halt the decline of House Sparrows by first assessing whether these species are also competing for quality sites. This can be achieved by adding specially designed nest boxes that prevent Common Mynas from entering. Public awareness campaigns, such as removal programs, are needed to change public perceptions of management efforts.

## Data Availability

Data is unavailable due to privacy.
